# Online verbal aggression on interpersonal trust among college students: the chain-mediating effect of core self-evaluation and emotional intelligence

**DOI:** 10.3389/fpsyt.2025.1556046

**Published:** 2025-06-20

**Authors:** Yao Yao, Xuejun Fan, Gang Chen, Ping Li, Song Liu

**Affiliations:** ^1^ School of Marxism, Hunan University of Chinese Medicine, Changsha, Hunan, China; ^2^ Institute of Higher Education, Hunan Academy of Education Sciences, Changsha, Hunan, China; ^3^ School of Education Science, Hunan Normal University, Changsha, Hunan, China; ^4^ College of Education, Zhejiang University, Hangzhou, Zhejiang, China; ^5^ Teacher Education College, Ningxia University, Yinchuan, Ningxia, China

**Keywords:** online verbal aggression, interpersonal trust, core self-evaluation, emotional intelligence, mediating effect

## Abstract

**Objective:**

This study aims to examine the effect of online verbal aggression on interpersonal trust and the chain-mediating role of core self-evaluation and emotional intelligence among college students.

**Method:**

A questionnaire survey was conducted among 869 college students with experiences of online verbal aggression using the Cyber Verbal Bullying Scale, Interpersonal Trust Scale, Core Self-Evaluation Scale, and Emotional Intelligence Scale.

**Results:**

(1) After controlling other latent variables, it was found that online verbal aggression had a significant direct negative effect on college students’ interpersonal trust. (2) Core self-evaluation and emotional intelligence played significant mediating roles in the relationship between online verbal aggression and interpersonal trust, and their combined chain mediation effect was also statistically significant.

**Conclusion:**

Victimization by online verbal aggression may lower core self-evaluation in college students, thereby impairing their emotional perception and regulation and ultimately reducing their interpersonal trust. Effective interventions should address self-evaluation, emotional intelligence, and interpersonal trust to mitigate the adverse effects of online verbal aggression on college students.

## 1 Introduction

The time spent at a university is the transition period between adolescence and early adulthood. The interpersonal relationships and mental health of college students can have a profound impact on their personal development following graduation (1). Interpersonal skills are indispensable for building and maintaining successful interpersonal relationships, among which interpersonal trust (IT) is the basis of communication and provides a stable psychological expectation for uncertainties in interpersonal interactions (2). Combining the different definitions of IT by Rotter and other scholars, IT can be described as the psychological expectation under which an individual considers the words, promises, and statements of others as generally reliable (3). Research has shown that IT is positively correlated with college students’ self-worth, control ability, and life satisfaction. IT has been proven to be an important factor affecting college students’ mental health and social adaptation (4).

The aggressive behavior of an individual refers to the act of intentionally hurting others against their will (5), which can damage the physical and mental health of both the aggressor and the victim and the social atmosphere. About one-third of college students show a moderate tendency to conduct aggressive behavior (6). According to its different forms, aggressive behavior can be divided into physical, relationship, and verbal aggression. Online verbal aggression (OVA) is an individual’s deliberate attack on others through words on the Internet. In contrast to ordinary offline verbal aggression, OVA can be anonymous, open to public, and free from temporal and geographical limits, and it usually remains for a certain period of time on the Internet, during which the aggressive words can still be browsed and responded to by the victims and others (7). The consequences may also be different from ordinary verbal aggression. Cyberbullying, which is dominated by OVA, has a wide range of negative social and psychological effects such as low self-esteem and difficulties in communicating with others (8). Virtual OVA may also extend to offline violence, leading to serious events on campus and even in society (9). Therefore, the influence of OVA on college students’ interpersonal communication requires further study.

Existing studies have shown a significant negative correlation between IT and aggressive behaviors (4). Core self-evaluation (CSE) and emotional intelligence (EI) were also related to IT and aggressive behavior, respectively. CSE, put forward by Judge and other scholars, is a basic value judgment held by individuals regarding themselves, the world, and others. This personality structure contains four personality traits: self-esteem (the individual’s personal feeling of his/her own value), general self-efficacy (the individual’s belief in his/her ability to achieve goals in a specific field), nervousness (low emotional adjustment ability and ease of experiencing negative emotions), and locus of control (the degree to which an individual thinks he/she controls the surrounding environment) (10). Research has shown that college students’ CSE can significantly positively predict IT (11), and CSE is negatively correlated with aggressive behavior (12). Emotional intelligence (EI) refers to the ability of individuals to identify, express, evaluate, and regulate their own emotions and the ability to use emotions to solve problems and promote cognitive activities (13), including emotional awareness, emotional feedback, and emotional adaptation. Previous studies have shown that EI is related to IT (14) and aggressive behavior (15) to varying degrees. There is also a positive correlation between CSE and EI (16).

According to a comparative study by Li et al. (17), traditional and online aggression share certain characteristics yet differ in their forms of expression and technological contexts. Consequently, OVA may also influence college students’ IT. CSE and EI may serve as key mediating variables in the relationship between OVA and IT. However, the extent to which OVA affects IT and the specific mediating roles of CSE and EI remain unclear. Therefore, this study investigated the pathways through which OVA impacts college students’ IT, with CSE and EI as potential mediators.

## 2 Theory-based assumptions and hypotheses

Cyberbullying is a growing problem because an increasing number of university students are using online interactivity, and online communication has become a tremendous part of their lives. OVA can make students susceptible to victimization throughout the day. Therefore, understanding the mechanisms supporting the path from OVA to IT is crucial to manage the negative influences of OVA on students, for them to maintain their mental health, and to help them develop trustworthy social relationships.

According to Shattered Assumption Theory, stressful life events can destroy individual belief systems and negatively impact mental health (18). OVA victimization can function as a source of stress and trauma by conveying information about the world that contradicts individuals’ prior assumptions. This disruption may lead to a breakdown in core beliefs, including diminished trust in others’ benevolence and reliability (19). Studies have found that self-esteem and emotions play key roles in the trauma process and affect trust.

**Figure 1:**
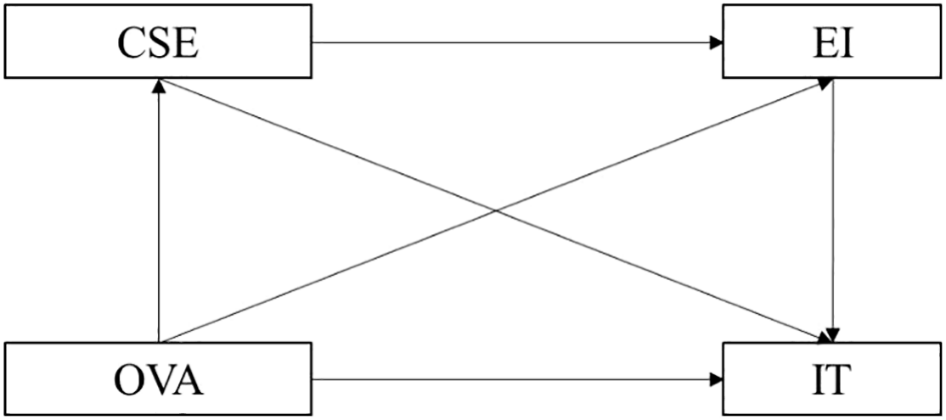
Theoretical model of OVA on IT. OVA, online verbal aggression; IT, interpersonal trust; CSE, core self-evaluation; EI, emotional intelligence.

Based on the analysis of the materials presented above, we adopted nine research hypotheses, as shown in **Figure 1**, namely:

H1. Online verbal aggression negatively affects interpersonal trust in college students.
H2. Core self-evaluation positively affects interpersonal trust in college students.
H3: Emotional intelligence positively affects interpersonal trust in college students.
H4: Online verbal aggression negatively affects core self-evaluation in college students.
H5: Online verbal aggression negatively affects emotional intelligence in college students.
H6: Core self-evaluation positively affects emotional intelligence in college students.
H7: Core self-evaluation mediates the relationship between online verbal aggression and interpersonal trust.
H8: Emotional intelligence mediates the link between online verbal aggression and interpersonal trust.
H9: Core self-evaluation and emotional intelligence together mediate the link between online verbal aggression and interpersonal trust.

## 3 Methods

### 3.1 Subjects

An online survey was conducted using convenience sampling among college students from four universities located in Hunan Province and Ningxia Province, China, who had experienced OVA. A total of 1,000 questionnaires were distributed, of which 932 were returned. After data screening, 869 questionnaires were deemed valid, resulting in a response rate of 86.9%. The final sample included 512 male and 357 female students with ages ranging from 17 to 29 years (M = 23.11, SD = 3.75), as detailed in **Table 1**.

**Table 1:** Characteristics of the respondents (*n* = 869).

Items	Cases (*n*)	Proportion (%)
Gender		
Female	357	41.08
Male	512	58.92
Age, years		
<18	63	7.25
18–20	312	35.90
21–23	346	39.82
>23	148	17.31
School enrollment		
Hunan Normal University	294	33.83
Hunan University	161	18.53
Hunan University of Chinese Medicine	270	31.07
Ningxia University	144	16.57

### 3.2 Survey instruments

#### 3.2.1 Measurement of online verbal aggressive behavior

OVA was assessed using the Cyber Victim and Bullying Scale developed by Cetin et al. (20). This scale comprises seven items rated on a five-point Likert scale, ranging from 1 (“never”) to 5 (“always”). Higher scores indicate more severe experiences of online verbal aggression. Previous studies have demonstrated the strong psychometric properties of the scale, with a reported Cronbach’s α coefficient of 0.91 and factor loadings for all items exceeding 0.68, indicating high reliability and construct validity (21). In the present study, Cronbach’s α coefficient was 0.86, further supporting the internal consistency of the scale.

#### 3.2.2 Measurement of interpersonal trust

IT was assessed using the Interpersonal Trust Scale developed by Rotter (3), which consists of 25 items addressing trust across various situations and social roles. Each item is rated on a five-point Likert scale, ranging from 1 (“completely disagree”) to 5 (“completely agree”). A total of 13 items were reverse-scored. The total score ranges from 25 to 125, with a median score of 75, with higher scores indicating a greater level of interpersonal trust. The Chinese version of the scale adapted by Wang et al. (22) has demonstrated good reliability and validity. Previous studies reported Cronbach’s α coefficient of 0.83 (23). In the current study, the Cronbach’s α coefficient was 0.94, indicating excellent internal consistency.

#### 3.2.3 Measurement of core self-evaluation

CSE was assessed using the Chinese version of the Core Self-Evaluation Scale originally developed by Judge and subsequently translated and revised by Wang (24). The scale consists of 10 items, six of which are reverse-scored. Each item is rated on a five-point Likert scale, ranging from 1 (“completely disagree”) to 5 (“completely agree”). The total score ranges from 10 to 20, with higher scores indicating higher levels of core self-evaluation. Previous validation studies reported a Cronbach’s α coefficient of 0.83 and demonstrated good criterion-related validity (24). In this study, the scale yielded a Cronbach’s α coefficient of 0.85, indicating strong internal consistency.

#### 3.2.4 Measurement of emotional intelligence

EI was assessed using the Chinese version of the Emotional Intelligence Scale developed by Schutte et al. and adapted and translated by Wang and He (25). The scale comprises 33 items, including three reverse-scored items. Each item is rated on a five-point Likert scale, ranging from 1 (“does not fit me at all”) to 5 (“fits me perfectly”). Higher scores indicate higher levels of emotional intelligence. Previous research has reported Cronbach’s α coefficient of 0.90 (26). In the present study, the Cronbach’s α coefficient was 0.91, demonstrating excellent internal consistency. All scales used in this study were administered with appropriate permission.

### 3.3 Statistical analysis

Data were analyzed using SPSS 26.0, and Mplus 8.0. SPSS software was used for descriptive statistics, correlation analysis, and common method bias testing. Mplus was used to test chain-mediating effects. Statistical significance was set as *P <*0.05.

## 4 Results

### 4.1 Common method bias testing

The Harman single-factor method was used to assess the common method bias. The results showed that 12 factors had characteristic roots greater than 1, and the variance explanation rate of the common factor with the largest characteristic root was 22.78%. Because this value is below the critical threshold of 40%, it indicates that no significant common method bias was present in this study.

### 4.2 Descriptive statistics and correlation analysis

Descriptive statistics and Pearson’s correlation analyses were performed on the total scores of the four latent variables: OVA, IT, CSE, and EI. The results indicated that all of the variables were significantly correlated with one another (*P* < 0.01). Specifically, OVA was negatively correlated with IT, CSE, and EI, whereas IT, CSE, and EI showed significantly positive intercorrelations (**Table 2**).

**Table 2:** Descriptive statistics and correlations of the latent variables of interest.

Variables	M ± SD	1	2	3	4
1. OVA	21.81 ± 6.05	1			
2. IT	74.47 ± 19.18	-0.441^**^	1		
3. CSE	33.95 ± 6.91	-0.326^**^	0.399^**^	1	
4. EI	107.58 ± 18.31	-0.396^**^	0.563^**^	0.392^**^	1

OVA, online verbal aggression; IT, interpersonal trust; CSE, core self-evaluation; EI, emotional intelligence.

^**^ *P* < 0.01 (two-tailed).

### 4.3 Online verbal aggression on interpersonal trust: chain mediation modeling

**Figure 2:**
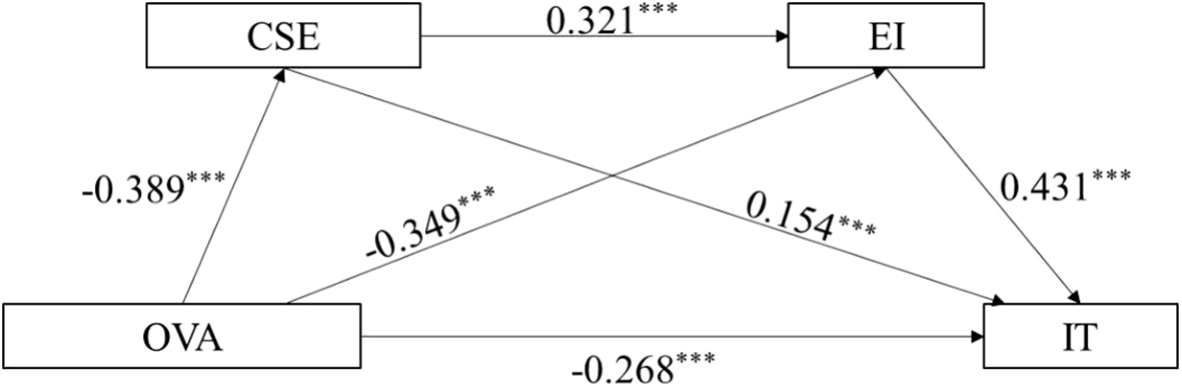
Chain mediation model of OVA on IT. OVA, online verbal aggression; IT, interpersonal trust; CSE, core self-evaluation; EI, emotional intelligence. ***: P<0.001.

A latent variable model with a chain mediation structure was constructed, in which OVA served as the independent variable, IT as the dependent variable, and CSE and EI as mediating variables. As shown in **Table 3**, the model fit indices indicated a good fit to the data: CFI = 0.923, TLI = 0.921, and RMSEA = 0.026 (95% CI: 0.025–0.028), which is consistent with the recommended thresholds (27). As presented in **Table 4** and **Figure 2**, the structural equation modeling results demonstrated that after controlling for other variables, OVA had a significant direct negative effect on IT (*β* = –0.268, *P* < 0.001). Both CSE (*β* = 0.154, *P* < 0.001) and EI (*β* = 0.431, *P* < 0.001) had significant direct positive effects on IT. OVA was also found to negatively affect CSE (*β* = − 0.389, *P* < 0.001) and EI (*β* = –0.349, *P* < 0.001), whereas CSE exerted a positive effect on EI (*β* = 0.321, *P* < 0.001). The mediation analysis revealed that the total standardized indirect effect of OVA on IT was −0.264 (*P* < 0.001). Specifically, the standardized indirect effect of CSE alone was –0.060 (*P* < 0.001), accounting for 22.7% of the total mediation effect. The indirect effect of EI alone was –0.151 (*P* < 0.001), contributing to 57.2% of the total effect. Additionally, the chain mediation effect via both CSE and EI was –0.054 (*P* < 0.001), representing 20.1% of the total effect. This chain mediation pathway was statistically significant, as shown in **Tables 5**, **6**.

**Table 3:** Model fit information.

Model fit index	*χ* ^2^	*χ* ^2^/*df*	CFI	TLI	RMSEA (95% CI)
Value	4,300.487	1.596	0.923	0.921	0.026 (0.025, 0.028)

**Table 4:** Standardized model results.

Variable model	Effect (*β*)	SE	*t*	*P*
OVA→IT	-0.268	0.039	-6.895	<0.001
CSE→IT	0.154	0.037	4.173	<0.001
EI→IT	0.431	0.042	10.161	<0.001
OVA→CSE	-0.389	0.039	-10.062	<0.001
OVA→EI	-0.349	0.039	-8.935	<0.001
CSE→EI	0.321	0.043	7.429	<0.001

OVA, online verbal aggression; IT, interpersonal trust; CSE, core self-evaluation; EI, emotional intelligence.

→ indicates the direction of path (the effect of the left on the right).

**Table 5:** Total, direct, and indirect links of online verbal aggression and interpersonal trust.

Effect (*β*)	SE	*T*	*P*	Bootstrap 95% CI	
				Lower	Upper
Total effect: IT on OVA					
-0.533	0.036	-14.599	<0.001	-0.604	-0.460
Direct effect: IT on OVA					
-0.268	0.039	-6.895	<0.001	-0.343	-0.191
Total indirect effect					
-0.264	0.027	-9.692	<0.001	-0.318	-0.210
Specific indirect effect 1: IT on OVA through CSE					
-0.060	0.015	-3.902	<0.001	-0.094	-0.033
Specific indirect effect 2: IT on OVA through EI					
-0.151	0.023	-6.511	<0.001	-0.198	-0.107
Specific (chained) indirect effect 3: IT on OVA through CSE and EI					
-0.054	0.011	-5.046	<0.001	-0.078	-0.036

OVA, online verbal aggression; IT, interpersonal trust; CSE, core self-evaluation; EI, emotional intelligence.

**Table 6:** Mediation analysis.

Effect	Path	Standardized effect value	*P*	Proportion
Total indirect effect		-0.264	<0.001	100%
Mediating effect 1	OVA→CSE→IT	-0.060	<0.001	22.7%
Mediating effect 2	OVA→EI→IT	-0.151	<0.001	57.2%
Chain-mediating effect	OVA→CSE→EI→IT	-0.054	<0.001	20.1%

OVA, online verbal aggression; IT, interpersonal trust; CSE, core self-evaluation; EI, emotional intelligence.

## 5 Discussion

Using latent variable modeling and analysis, this study examined the influence of OVA on college students’ IT with a particular focus on the chain-mediating roles of CSE and EI. The results indicated that OVA significantly and negatively predicted IT among college students, suggesting that exposure to OVA diminished their levels of interpersonal trust. This finding aligns with previous research on the relationship between aggression and trust (4).

OVA, as a form of virtual aggression, shares many characteristics with traditional aggression but also exhibits unique features such as anonymity and public exposure. Despite these distinctions, there remains limited empirical evidence regarding the relationship between OVA and IT, particularly concerning whether its impact parallels that of conventional forms of aggression. Based on a survey of Chinese college students, this study found that OVA occurring in online environments can significantly affect students’ trust in real-life interpersonal interactions.

According to Schultz’s Situational Trust Model, trust is fundamentally shaped by whether a trusted individual fulfils the positive expectations of the trustor. Furthermore, the effects of media usage and satisfaction on individual attitudes are complex and multifaceted (28). Given the Internet’s characteristics of unreality, fragility, freedom, and anonymity, verbal aggression in virtual spaces can exert even greater psychological pressure and trauma on individuals, particularly college students. This, in turn, may undermine their ability to engage in open and trusting communication. Therefore, OVA warrants greater research and practical attention, as it is often difficult to detect and intervene. Preventing the adverse impacts of OVA on students’ mental and social well-being remains a critical issue for educational institutions.

This study demonstrated that CSE and EI jointly serve as chain mediators in the relationship between OVA and IT. Specifically, OVA negatively influenced CSE, which, in turn, reduced EI and ultimately lowered IT. Core self-evaluation reflects an individual’s sense of self-worth and social acceptance and serves as a reference for engaging in interpersonal relationships (29). On one hand, experiences of aggressive behavior may reduce self-esteem and lead to negative interpersonal beliefs. However, being a victim of OVA and experiencing low self-evaluation may stem from inadequate acceptance by peer groups. This suggests a potentially complex interplay among OVA, CSE, and IT.

Research has shown that individuals with high CSE are better able to self-regulate and establish strong social connections as they possess higher emotional intelligence and are more capable of perceiving and managing their own and others’ emotions (16). Conversely, those with low CSE may struggle with emotion regulation. Drawing on previous research and the findings of this study, we conclude that CSE and EI partially mediate the relationship between OVA and IT. Victims of OVA may develop lower self-evaluations, which impair their emotional perception and regulation, ultimately reducing their interpersonal trust. These findings suggest that higher education professionals can intervene in preventing the harmful effects of OVA by targeting this psychological pathway. Educational institutions should consider OVA victimization seriously, provide support to affected students, and help them rebuild a healthy sense of self. Additionally, promoting responsible Internet behavior and enhancing emotional intelligence through campus-wide education could serve as an effective preventive measure.

This study confirmed that CSE and EI independently played partial mediating roles in the relationship between OVA and IT, with both paths showing statistically significant effects. This finding supports earlier research on traditional aggression and interpersonal trust and further highlights the similarity in the consequences of OVA and conventional aggression.

This study has several limitations. First, it employed a cross-sectional design based on self-reported survey data, which restricts its ability to track changes over time and infer causality. Future research should adopt longitudinal or experimental designs to verify these results and explore the impact of the developmental dynamics of OVA on IT. Second, although CSE and EI were identified as mediators in this study, other variables such as personality traits or Internet use patterns may also play important roles in the relationship between OVA and IT. Future studies should consider these additional factors and examine the mechanisms by which OVA influences IT across demographic groups, personality profiles, and cultural settings. This would support the development of tailored and effective interventions to prevent and mitigate the effects of OVA among college students.

## 6 Conclusion

Online verbal aggression may reduce college students’ core self-evaluation, thereby impairing their ability to perceive and regulate emotions, which diminishes their interpersonal trust. Understanding this underlying mechanism provides valuable insights to develop targeted interventions and preventive strategies to mitigate the negative impacts of online verbal aggression on college students.

## Data Availability

The raw data supporting the conclusions of this article will be made available by the authors, without undue reservation.
